# Fruit Wine Obtained from Melon by-Products: Physico-Chemical and Sensory Analysis, and Characterization of Key Aromas by GC-MS

**DOI:** 10.3390/foods11223619

**Published:** 2022-11-13

**Authors:** José Ángel Salas-Millán, Arantxa Aznar, Encarnación Conesa, Andrés Conesa-Bueno, Encarna Aguayo

**Affiliations:** 1 Postharvest and Refrigeration Group, Universidad Politécnica de Cartagena (UPCT), 30202 Cartagena, Spain; 2 Food Quality and Health Group, Institute of Plant Biotechnology, Universidad Politécnica de Cartagena (UPCT), Campus Muralla del Mar, 30202 Cartagena, Spain; 3 JimboFresh International SLL, C/Mina Buena Suerte, 1, 30360 Murcia, Spain; 4 Department of Agronomical Engineering, Institute of Plant Biotechnology, Universidad Politécnica de Cartagena (UPCT), Paseo Alfonso XIII, 48, 30203 Cartagena, Spain

**Keywords:** cosmetic defects, beverage, aroma, waste, food loss, fermentative process

## Abstract

About 20% of fresh fruits and vegetables are rejected for not meeting the superficial aesthetic standards (color, shape, and size). Part of the food production is not used in the human food chain. The transformation of these fresh products into novel re-valuable ones is a challenge for a sustainable food industry. This research studies an alcoholic fermentation fruit-based wine from two melon (*Cucumis melo* L.) cultivars: Jimbee^®^ (smooth and yellow skin with orange flesh) and Okashi^®^ (netted yellow-orange skin with pale green flesh). The melon juice (must) was fermented by *Saccharomyces cerevisiae* and enriched in sucrose and organic acids to achieve alcoholic fermentation, acidity, and flavors, obtaining a fruity-flavored and dry melon-based wine with 10° alcoholic grade, in both melon cultivars. The volatile compounds were measured by GC-MS and the odor activity value (OAV) was calculated. The Jimbee and Okashi melon wines increased their aromatic profile due to an increment in medium-chain fatty acid ethyl esters such as ethyl hexanoate, ethyl octanoate, and ethyl decanoate (OAV > 1), which contributed to the fruity aroma. Other volatile compounds such as ethyl 9-decenoate and phenethyl acetate (OAV > 1) appeared in the Okashi wine, which brought a floral aroma. For sensory evaluation (40–100), the Jimbee cultivar, with its orange flesh, scored 68.2 and the Okashi cultivar, with pale green flesh, scored 82.8, which was the preferred melon-based wine. This is an example of a circular economy model to produce a fruit-based wine with commercial potential and satisfactory sensory evaluation.

## 1. Introduction

In recent times, supermarkets and even consumers have come to reject fruits and vegetables as marketable fresh products due to merely superficial cosmetic imperfections [1]. Cosmetic quality standards for fruits and vegetables are specific requirements regarding color, shape, and size that harvested products need to meet after preparation and packaging. This means that part of the food production will not be used in the human food chain but will instead be intended for low-value valorization. In general, these cosmetic quality standards are often linked to food losses (if they occur before reaching the food chain) or food waste (at the end of the food chain as retail and final consumption). This food loss and/or waste has a significant environmental impact (land use, water consumption, greenhouse gas emissions, etc.) and financial implications. For this reason, reducing food loss and waste has been identified as an important means of achieving future sustainability goals [2]. It is a challenge for the agricultural sector to stimulate a circular economy model where these fruits and vegetables bearing superficial cosmetic imperfections can be revalued into novel food products such as fruit beverages and fruit-based wine by alcoholic fermentation. Winemaking is traditionally based on using grapes as the fruit of choice, although there are also traditional examples of fermented drinks made from rice, honey, and other fruits, such as persimmon and kiwi [3,4]. Several fruits are grown in huge quantities around the world to obtain alcohol in the fermenting process. The process is similar to grape-based winemaking through alcoholic fermentation using yeasts, *Saccharomyces cerevisiae* as the predominant species, obtaining ethanol, CO_2_, and other secondary metabolites that increase the aroma profile such as esters, and higher alcohols [5]. Fruits other than grapes such as apples, berries, cherries, wild apricots, kiwifruit, plums, peaches, and strawberries are used in winemaking in many parts of the world. Cider, or apple wine, is obtained from fermented apples and is one of the most popular non-grape fruit wines [6]. In the US and Canada, some examples of fruit-based winemaking include strawberries, plums, peaches, blackberries, etc. Europe predominates in the use of apples and peaches, whilst Asia includes tropical and subtropical fruits such as mango, cocoa, pineapple, etc. [7]. The main components of theses fruits wines (alcohols, monoterpenic compounds, and ethyl esters) resulted in the production of fruity, green apple, banana, sweet, citrus, roses, and honey aroma, whereas aromas from typical white wines by grapes are described as fruity, floral, green, sweet, and fatty [7]. Melon (*Cucumis melo* L.) is a fruit with a great commercial value that is cultivated in different parts of the world due to its adaptability to many soil types and temperatures.

Melon production has risen by 20 million tons in the world [8]. Spain was the world’s foremost melon exporter in 2020, producing more than 600,000 tonnes and exporting 440,000 tonnes, approximately 20% of total global exports [9]. *C. melo* cultivars belonging to different botanical varieties such as cantalupensis, reticulatus, inodorus etc., are highly valued for their sweetness, aroma, and pulp texture. Some authors studied the physicochemical analysis of melon-based wine [10] and evaluated the majorities of volatiles in melon distillates [11]. In this research, we study two melon cultivars for fruit-based winemaking: Jimbee^®^ (orange flesh) and Okashi^®^ (pale green flesh); no references for these fermented wines are available. The objective of this study is to develop and characterize a fruit-based wine from two melon cultivars with different flesh colors, when those fruits are discarded by the supermarket as fresh fruits for not meeting the required aesthetic standards, despite still having a high sensorial quality that could provide a newly fermented wine with commercial potential.

## 2. Materials and Methods

### 2.1. Small-Scale Melon Wine

**Figure 1:**
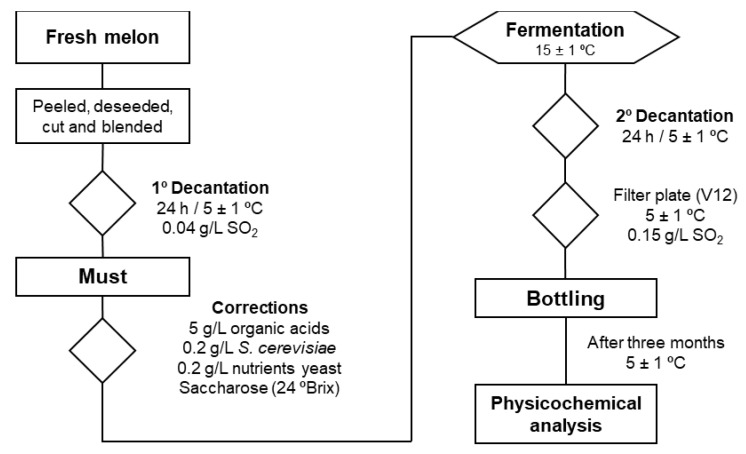
Scheme of the production of melon wine.

Two commercial cultivars, Jimbee^®^ (smooth and yellow skin with orange flesh, honeydew melon type) and Okashi^®^ (netted yellow-orange skin with pale green flesh, galia melon type) were obtained from JimboFresh International Coop. (Murcia, Spain) in the summer season. These melons did not reach the aesthetic standards required by supermarkets, in spite of being healthy fruit but with a small caliber and some sunspots. Fresh melons were transported by car to the laboratory (30 km). Fruits were hand-washed, peeled, deseeded, and cut into small pieces before blending in a commercial blender (OK Juicer OPJ 4321) to obtain the juice to be used as a must (Figure 1). The decantation and fermentation processes were run in 2 L closed jars by triplicate, with an airlocker to avoid the entrance of air. The musts were decanted for 24 h at 1 °C and 40 mg/L of SO_2_ was added. Both musts were decanted at 5 °C, and the liquid obtained was used for alcoholic fermentation, after adding 5 g/L of tartaric acid and malic acid (1:1, *w*/*w*), 0.2 g/L of commercial yeast (Zymaflore^®^ X5, Laffort, Bordeaux, France), 0.2 g/L of nutrient yeast (Superstart^®^ Blanc, Laffort, Bordeaux, France), and commercial saccharose until the must reached 24 °Brix. Alcoholic fermentation was run at 15 °C until the total soluble solids (TSS) stabilized. At that point, the TSS obtained was 11.30 ± 0.31 °Brix in the Jimbee and 8.93 ± 0.07 °Brix in the Okashi wines. Finally, after a second decantation at 5 °C, SO_2_ was added to the melon wines until it reached 150 mg/L, filtered through plate (Filtro Jolly 20, MORI, Tavarnelle Val di Pesa, Italy) using V12 filter sheets 20 × 20 (Gruppo Cordenons SpA, Milano, Italy). The wines were then put into glass bottles (750 mL) and stored at 5 °C for 3 months. Three replicates were prepared per melon cultivar. Melon-based wine was making by triplicate with three different batches.

### 2.2. Physicochemical Analysis

Physicochemical parameters—alcohol strength (% *v*/*v*), TSS, extracts, and total and free SO_2_—were measured using the standardized method by the International Organization of Vine and Wine [12] in the musts and the melon wines. The pH was measured using a pH meter (Crison 501, Barcelona, Spain). Total acidity (TA), and volatile acidity (VA) were determined with a titrator acidity (T50, Metter Toledo, Milan, Italy), and expressed as gram equivalent citric acid per L of must (g CE/L) and gram equivalent tartaric acid per L of wine (g TE/L) for TA and gram equivalent acetic acid per L of wine for VA. Color was determined using a colorimeter (Chroma Meter CR–400, Minolta, Tokyo, Japan) previously calibrated with a white reference plate. A glass cuvette with 10 mm optical thickness was used to measure the must or the melon wine CIELAB parameters. Three color readings were taken per sample. Hue angle (°h), a qualitative attribute of color, was calculated as tan^−1^ (b*/a*). The value of chroma (C*), which is the degree of quantitative difference of h° with reference to grey, was evaluated as (a*^2^ + b*^2^)^1/2^.

### 2.3. Individual Soluble Sugar by Ionic Chromatography (IC)

Individual soluble sugars were determined according to Hu et al. [13], with a slight modification. A Metrohm 871 Advanced Compact IC (Metrohm, Ionenstrasse, Switzerland) was used. The IC separation was carried out on a Metrosep Carb 1 column (5 μm, 150 × 4.00 mm) with a Metrosep guard column, NaOH 80 mM as eluent, and the flow was set at 1 mL/min and the oven temperature at 32 °C. The analytes were analyzed with an amperometry detector, working and reference electrodes were made of gold and platinum, respectively, and the potential range was set for ±2.00. Residual sugar in the melon wine was calculated as the sum of glucose, fructose, saccharose, and maltose, and the sugar-free extract (g/L) as the difference between the dry extract and the residual sugar.

### 2.4. Determination of Total Polyphenol Content (TPC) and Antioxidant Capacities (FRAP and TEAC)

Melon wine samples were diluted in water (1:5) and their total polyphenol content (TPC) and antioxidant capacities were determined, using a multiscan plate spectrophotometer (Tecan infinite M200, Männedorf, Switzerland). For TPC, Folin-Ciocolteau’s reagent was used and performed as detailed by Martínez-Sánchez et al. [14], using gallic acid as the standard (mg gallic acid equivalent/L of must or wine, mg GAE/L). Ferric reducing antioxidant capacity (FRAP) was measured by the increment of absorbance at 593 nm due to the reduction of ferrous cation Fe + 3 by antioxidants at 37 °C and pH 3.6 [15] and was evaluated according to cation ferrous Fe + 2 linear calibration (mmol Fe^+2^/L of wine). Trolox equivalent antioxidant capacity (TEAC) was performed according to [16], measuring the reduction of radical ABTS at 734 nm, and Trolox was used as the equivalent standard, with data expressed as mmol Trolox equivalent/L of wine (mmol TE/L of wine).

### 2.5. Analysis of Volatile Compounds by GC-MS

Headspace solid-phase micro-extraction (HS-SPME) was used to extract the volatile profiles from the samples [3], which were then identified using gas chromatography (Agilent 7890B) coupled to a mass spectrometer (Agilent MSD 5977A), with an autosampler (Gerstel MPS 2XL Twister, Linthicum Heights, MD, USA). A 15 mL SPME glass vial containing 1 g of sodium chloride and 3-octanol as internal standard (i.s.) (20 μg/mL in sample, Sigma–Aldrich, St. Louis, MO, USA) was filled with 7.5 mL of sample supernatant. The sample was incubated at 40 °C for 10 min to equilibrate before volatile chemicals were extracted using HS-SPME with a DVB/CAR/PDMS fiber (50/30 μm, Supelco, Bellefonte, PA, USA) inserted in the vial’s headspace for 45 min (40 °C). The conditions detected by the GC–MS were based on Lu et al. [3]. Compounds were separated using a VF-WAXms (30 m × 0.25 mm, i.d. 0.25 μm), carrier gas (e.g., He) velocity at 1 mL/min, splitless mode, and 240 °C injection mode. The oven temperature program was initially set at 40 °C to 100 °C by 2 °C/min, then raised to 250 °C by 10 °C/min and held for 10 min. For MS conditions, the electron impact (EI) was set at 70 eF, the source temperature at 230 °C, and the scan time segments ranged from 35 to 550 m/z.

The NIST database was used to identify volatile chemicals by comparing the mass spectrum and retention index (RI) by the Kovats Index (KI). RI values were calculated using n-alkane external standard solution C8-C20 (Sigma-Aldrich, St. Louis, MO, USA) with the same GC-MS conditions. The ratio of the GC peak area of each volatile in the total ion chromatogram to the peak area of the internal standard was used to determine the concentration of the analyses as a semi-quantification, as per the following equation [3,17], where I.S. is the internal standard.

$$Volatile\;compound\;\left(\mu g/L\right)=\frac{Peak\;area\;of\;unknown\cdot \;Concentration\;I.S.\;\left(\mu g/L\right)}{Peak\;area\;of\;I.S.}$$

### 2.6. Odor Activity Value (OAV) and Relative Odor Contribution (ROC)

Odor activity values (OAV) and relative odor contributions (ROC) are both conventional markers used for quantifying the sensory contribution of aromatic chemicals to wine flavor [18]. OAV was calculated by dividing a compound’s mean concentration (n = 3) by its odor threshold value, as previously reported by other authors [18,19,20] The ROC of each aroma component is measured as the ratio of the compound’s OAV to the overall OAV of each wine.

### 2.7. Sensory Evaluation

The sensory evaluation was carried out in a normalized tasting room (at 22 °C) and conducted in standardized random coded wine glasses with 15 mL of melon wine, and both varieties were described separately. Twelve judges (7 women and 5 men, between 30 and 55 years of age) of the research group formed the sensory panel. Each judge analyzed two duplicates per wine and the sensory evaluation assessment was performed according to OIV 332A/2009 resolution [12], where judges evaluated different attributes (Table 1). Each sensory attribute’s score was recorded, and an overall score was calculated by summing the individual attribute ratings.

## 3. Results and Discussions

### 3.1. Must Composition

Physicochemical Jimbee and Okashi musts parameters are shown in Table 2. Initially, the pH was 6.28 and 6.05, TA 1.43 and 1.96 g CE/L for the Jimbee and Okashi melon musts, respectively. These values are in the range of other fresh melon cultivars recently reported [21]. The h° was 65° for the Jimbee and 109° for the Okashi musts, due to its typical orangeness, and pale green-slightly yellow flesh color, respectively. After must correction, where organic acids, yeast, nutrients, and saccharose were added, the pH decreased to almost 4.5. In both melon musts, the addition of saccharose reached 24 °Brix, being monitored to know the evolution of alcoholic fermentation. After fermentation, in the first week, the TSS dropped to 12–15 °Brix and slowly decreased in the following week, staying stable at 11.3 and 8.9 °Brix for the Jimbee and Okashi wines, respectively. The sugar stability indicated the end of the alcoholic fermentation.

### 3.2. Melon Wines Characterization

The results of the physico-chemical analysis for melon wines are shown in Table 3. In both melon wines, the alcohol content at the end of fermentation was closed to 10°. The TSS were 11.30 and 8.93, °Brix and residual sugar were 1.82 and 1.67 g/L for the Jimbee and Okashi wines, respectively. These results characterize both melon beverages as dry wine (<4 g/L in residual sugar) [12]. Both melon wines presented a similar amount of individual soluble sugars (Table 4), with fructose being the most abundant soluble sugar (1.02 and 1.44 g/L), followed by saccharose in the Jimbee wine (0.63 g/L), but it was not detected in the Okashi wine. Maltose and glucose were quantified in very low concentrations (Table 3). However, the sugar-free extract was 53.38 and 25.33 g/L for the Jimbee and Okashi wines; this is an important wine parameter when evaluating fullness and harmony; dry white wines are usually below 25 g/L [18].

The pH dropped to close to 4 in both melon wines, which is slightly higher than the usual recommendation for the stabilization of wines (pH between 3.1 to 3.6) [22]. The TA was higher in the Jimbee melon wine than in the Okashi, 8.86 and 6.88 g TE/L, respectively. In general, table wines usual ranges from 5.5 to 8.5 g TE/L, but white wine is preferred with a slightly higher TA than red wines [22]. These results suggest that these melon wines can reach similar acidity to that demanded in white grape wines, depending on the quantity and type of organic acids added. Total SO_2_ was 142.4 and 97.6 mg/L for the Jimbee and Okashi wines, respectively. According to the OIV, the total SO_2_ must be lower than 150 mg/L for red wines and lower than 200 mg/L for white wines. The free SO_2_ was 131.84 mg/L in the Jimbee and 63.15 mg/L in the Okashi melon wines. SO_2_, used as an antioxidant, enzyme inhibitions, and antimicrobial activity, all exist in different states in wine as bounded or free, and these bonds are reversible or irreversible with major constituents of wine (carbonyl compounds, pyruvic acid, α-ketoglutarate, sugars, sugar acids) [23]. Free SO_2_ is the most easily absorbed by the microorganism, values of 0.8 to 1.5 mg/L are enough to suppress wild yeast and bacteria, and it decreases during the time in the bottle [22]. For total SO_2_, the typical value for white wines is between 37 and 59 mg/L [18], and for fruit-based (peach, blueberry, bayberry, dragon fruit, Chinese quince) wines. [24] reported a range from 22 to 51 mg/L for total SO_2_ and between 3.2 and 13 mg/L for free SO_2_. Our results comply with the current regulations for white grape wines, suggesting that our melon wines are below the limits of total SO2 and with a proper content of free SO_2_ for stabilizing melon wine for a long time. Table 4 shows the results of the color evaluation. The L* was 50.17 for the Jimbee melon wine, and 61.32 for the Okashi wine. As expected, the coloration of the melon flesh provided lower luminosity values than those found in white wines obtained from grapes (<95) [25,26]. As mentioned before, the Jimbee melon must was characterized by orange flesh (°h = 65.46) and with the fermentation process, the final wine color changed to yellow (°h = 95.67), where most of the natural pigments (carotenoid) sedimented and were removed by filtering. However, chroma values measured in the Jimbee must and the melon wine were quite similar, 15.82 and 13.68, respectively. The °h for the Okashi melon scarcely changed, ranging from 109 in the must to 107 in the wine, with a slight trend from greenish color to yellow (Table 2 and Table 4). These color parameters obtained in melon wines were similar to those for white grape wines [25,26]. However, both the melon wines had lower lightness, but that can be increased by using a different type of filtering [27].

### 3.3. Total Polyphenol Content (TPC) and Antioxidant Capacities (FRAP and TEAC)

The TPC and antioxidant capacities measured in the Jimbee and Okashi musts were similar to those found in other studies performed in fresh melon [28,29]. The effects of the alcoholic fermentation during fruit winemaking in TPC and antioxidant capacities are shown in Table 1 and Table 3. During the winemaking process, both melon cultivars showed the same trend, with TPC increasing by about 13%, duplicated FRAP level, and decreasing TEAC value. These differences between antioxidant assays (FRAP and TEAC) are due to the different chemical reactions that each assay involves. The FRAP method constitutes an approach based on electron transfer, specifically based on the reducing action of antioxidants. In this method, iron in the oxidized form of Fe^+3^ is reduced to Fe^+2^ at acidic pH [30]. TEAC measures the relative ability of antioxidants such as Trolox to scavenge the ABTS generated in the aqueous phase, the assay is based on a quencher of peroxyl radicals [31]. Furthermore, this difference in both antioxidant assays could be due to the relative differences in the phenolic transformation that occur during the winemaking process. [32] reported the same trend in antioxidant capacity between the must to wine process (13%).

Jimbee and Okashi wines exhibited TPC levels of 406 and 242 mg GAE/L, in the range of the 350 mg GAE/L reported by Minkova et al. [10] in melon wines. These results were higher than in apple wine (223 mg catechin equivalent/L) but lower than in blackcurrant (420 mg catechin equivalent/L), principally because that fruit is rich in anthocyanins, thus increasing its TPC and antioxidant capacity [33]. Nevertheless, when we compare the TPC of melon wines versus Sauvignon blanc wines (242–278 mg GAE/L), the results are higher than or similar. Concerning the antioxidant capacity (TEAC), the melon wines presented 1.60 and 1.80 mmol TEAC/L; these levels were slightly higher than for Sauvignon blanc wines (1.26–1.50 mmol TEAC/L) [18].

### 3.4. Volatile Compounds in Melon Wines

The volatile compounds identified in these melon wines (Table 5) were classified as esters (34), alcohols (17), unsaturated aliphatics (3), aldehydes (5), organic acids (3), and miscellaneous (5). As expected, during the fermentation process, in both melon wines, the concentration of total volatile compounds increased. These increases went from 2145 μg/L, measured in the must, to 12,788 μg/L for the wine from the Jimbee melon, and from 1769 obtained in the must to 20,504 μg/L in the wine from Okashi. As expected, the alcoholic fermentation produced CO_2_, ethanol, acetic acid, and other volatile compounds described below. The Jimbee must presented mainly ester compounds such as butyl acetate, ethyl acetate, isobutyl acetate, methyl acetate, and fatty alcohols as 1-octanol, 1-hexanol, and isomers of nonen-1-ol isomer. These same volatiles have been reported in orange honeydews and cantaloupe fresh melon [34,35]. At the end of the alcoholic fermentation, the increase in volatile content changed the aroma profile. The relative proportions of alcohol increased from 18% to 56%, mainly caused by the presence of ethanol, 3-methyl-1-butanol, 2,4-bis(1,1-dimethylethyl)-phenol, and phenylethyl alcohol produced by *S. cerevisiae* [5,36]. The content in the total ester volatile group also increased during fermentation, from 1021 to 4325 μg/L, decreasing the initial ester compounds to medium-chain fatty acid (MCFA) ethyl esters such as ethyl octanoate, ethyl decanoate, ethyl laurate, and ethyl hexanoate. However, other esters such as ethyl acetate and 2-methylbutyl acetate showed increases produced by *S. cerevisiae* [5] and which are commonly present in white and red wines [18,37].

With regard to the Okashi melon wine, acetate esters initially predominated as ethyl acetate, isobutyl acetate, butyl acetate, 2-methyl-1-butyl acetate and hexyl acetate, and aldehyde as 6-nonenal and 2,6-nonadienal; all of these had been previously described in muskmelon and Galia melon [35,38]. The content in alcohol compounds was lower than in the Jimbee cultivar, in which nonen-1-ol isomers were predominant in the Okashi must. As happened in the Jimbee wine, the relative proportion of volatile compounds in the Okashi must changed after alcoholic fermentation, the alcohol group increased with the same compounds described in the Jimbee wine but accompanied by an increase in benzyl alcohol (12.38 in must to 344.52 μg/L in wine) which was only found in the Okashi melon wine. The ester group profile also changed during fermentation, except for ethyl acetate and 2-methyl-1-butyl acetate. Other acetate esters quantified in the must but not identified in the wine were ethyl propionate, n-propyl acetate, isobutyl acetate, ethyl butanoate, ethyl 2-methyl-butanoate, butyl acetate, propyl 2-methyl-butanoate, pentyl acetate, hexyl acetate, 4-hexen-1-ol acetate, and phenylmethyl acetate. Instead, fatty acid esters predominated in the Jimbee and Okashi wines as ethyl octanoate and ethyl hexanoate, and other different compounds such as ethyl 9-decenoate and ethyl palmitate, usually described in Chardonnay wine [20] and other fruit-based wine developments such us persimmon and kiwi [3,4]. Two aromatic volatiles were quantified in both melon wines: bis(1,1-dimethylethyl)-benzene and 2,4-bis(1,1-dimethylethyl)-phenol, which have been reported in other alcoholic beverages such as white grape wine and rice wine [36,39]. Octanoic acid and acetic acid were the principal organic acids identified in both melon-based wines. Acetic acid, an organic acid that usually appears during alcoholic fermentation, had the largest quantities in the volatile profile in both the Jimbee and Okashi melon wines, which confirms the volatile acidity values obtained in the physicochemical analysis (Section 3.2). As mentioned above, the volatile profile of both melon-based wines was relatively similar to that of other grape-based wines described before, which could be reminiscent of grape wine, but with some features of typical melon aroma.

### 3.5. Odor Activity Value (OAV) and Relative Odor Contribution (ROC)

Table 6 shows the OAV values and ROC index of individual volatile compounds which contributed at the minimum threshold (OAV > 1) for its identification. Only six compounds from the 67 identified exceeded the threshold values in melon-based wines, the majority group corresponded to fatty acid ethyl ester usually produced by *S. cerevisiae* during alcoholic fermentation; it possesses a lower threshold value and provides fruity, candy, and perfume-like aroma [40]. Ethyl hexanoate had the highest OAV (19.62 in the Jimbee melon wine and 50.66 in the Okashi wine) and the contribution (ROC) of aroma in both wines was 0.68 and 0.44 in Jimbee and Okashi, respectively, which enhanced the fruity and wine-like aroma. Ethyl octanoate and methyl decanoate were quantified in both melon wines, and the ethyl hexanoate gave the wine a fruity aroma. Ethyl butanoate was found only in the Jimbee melon wine (OAV = 1.81), this compound is associated with pineapple- and apple-like aromas. The ethyl 9-decenoate and phenethyl acetate were concentrated enough to reach the minimum threshold in the Okashi melon wine, 6.96 and 1.04, respectively, both of which contributed to a flower-like aroma.

### 3.6. Sensory Evaluation

The sensorial wine quality is given in Table 7. The Jimbee melon wine obtained a total score of 68.17, whilst the Okashi wine obtained 82.33. In relation with the visual parameters, the limpidity and aspect of the Jimbee wine were lower than the Okashi, due to the presence of more particles in suspension, and as explained above, these two parameters were correlated to lightness color measurements (Section 3.2). Both melon wines had an average intensity and a good impression of quality in nose. However, the Jimbee melon wine had a lower genuineness than the Okashi wine, due to identified viticulture-based defects (e.g., raw material, some volatiles such as phenol, esters and oxido-reduction compounds, etc.), and the high content of volatile acidity obtained in the Jimbee wine (Table 4) [12]. These last differences were also found in the taste, where the panelists valued the Okashi melon wine to be better than the Jimbee wine, with a low intensity of defects, strong intensity, good persistence, and good-to-very good impression of quality according to the description detailed in OIV. These values are correlated with the higher volatile content measured in the Okashi melon wine, mainly the highest esters compound content with a fruity-like aroma (Table 6). Regarding the overall judgment, the Jimbee melon wine was judged as satisfactory, whilst the Okashi melon wine was close to receiving a very good general impression. Finally, the evaluated total scores defined the Okashi cultivar (82.83) as being a better melon-based wine which could be classified as Silver in accordance with OIV, mainly due to its better scores in visual and taste values, with this cultivar being preferred for melon-based wine.

## 4. Conclusions

In this research, fruit-based wines from two melon cultivars, Jimbee (orange flesh) and Okashi (pale green flesh), were obtained using melons discarded due to their cosmetic defects. Prior to alcoholic fermentation, with *S. cerevisiae*, the must was adjusted with organic acid (malic and tartaric acid) and saccharose to 24 °Brix to obtain a fruit-based dry wine with a 10° alcoholic grade in both development wines. During fermentation, both wines increased principally in MCFA ethyl esters such as ethyl hexanoate, ethyl octanoate, and ethyl decanoate, which are responsible for the aromatic profile of the wine. Melons with typical orange color flesh such as the Jimbee cultivar provided light-yellow color wine with a fruity aroma due to ester with OAV > 1, such as ethyl butanoate, ethyl hexanoate, ethyl octanoate, and ethyl decanoate. Melons with pale green color flesh such as the Okashi cultivar, provided melon wines with a light-yellow color with a fruity and floral aroma, characterized by the presence of ethyl hexanoate, ethyl octanoate, ethyl decanoate, ethyl 9-decenoate, and phenethyl acetate. In both melon wines, ethyl hexanoate had a high OAV score and accordingly, the predominant aroma, obtaining 19.62 for the Jimbee wine and 50.66 for the Okashi wine. In the sensory analysis, the Jimbee melon wine obtained a total score of 68.17, whilst the Okashi wine scored 82.83. These differences were found principally in the visual parameters and taste. Orange flesh melons have more particles in suspension which means lower limpidity and luminosity, both are parameters that are demanded in white grape wines. According to OIV categories, the Okashi melon would obtain the highest score, being classified as a silver level. However, these fruit wines do not have to follow the same standards required of traditional white grape wines, which guarantees their commercialization. In summary, this research is an example for the agricultural sector to stimulate a circular economy model using the by-products of the fresh melon industry to produce a novel fruit-based wine, rich in and TPC and antioxidant compounds, with economic potential in the market. This type of wine presents low carbon emissions and water footprints when discarded melon fruit is used.

## Figures and Tables

**Table 1:** Organoleptical characteristic and definition in sensory evaluation.

Organoleptical Characteristic	Definition	Range (Excellent to Inadequate)
**Visual:** Discrimination of differences in outside world with sensory impressions from visible light rays.		
Limpidity	Measure of cloudiness.	(5–1)
Aspect other than limpidity	Determine the full spectrum of visible properties of a product	(10–2)
**Nose:** Sensations perceived by the olfactory organ when stimulated by certain volatile substances.		
Genuineness	Measure degree of sensation perceived (magnitude) by the nose, of a viticulture, oenological defect of product	(6–2)
Positive intensity	Degree (magnitude) of full spectrum of qualitative odors perceived by nose.	(8–2)
Quality	Spectrum of properties and characteristics of a wine that gives an aptitude to satisfy nose, implicit or expressed needs	(16–8)
**Taste:** Full spectrum of sensations perceived with wine mouthfeel.		
Genuineness	Measure degree of sensation perceived (magnitude) by the taste, of a viticulture, oenological defect of product	(6–2)
Positive intensity	Degree (magnitude) of full spectrum of qualitative odors perceived by taste.	(8–2)
Harmonious persistence	To measure the length of residual olfacto-gustatory sensation, corresponding to the sensation perceived when the product is in mouth and length of time is measured.	(8–4)
Quality	Degree (magnitude) of full spectrum of qualitative odors perceived by taste	(22–10)
**Harmony—Overall judgement:** Corresponds to overall appraisal of a product.		(11–7)

**Table 2:** Physicochemical parameters of the Jimbee and Okashi melon musts.

Parameter	Jimbee Must	Okashi Must
TSS (°Brix)	11.30 ^z^ ± 0.31	8.93 ± 0.07
pH	6.28 ± 0.02	6.05 ± 0.00
TA (g CE/L)	1.43 ± 0.01	1.96 ± 0.03
L*	34.73 ± 0.18	39.23 ± 0.25
°Hue	65.46 ± 0.44	109.00 ± 0.48
Chroma	15.82 ± 0.17	20.65 ± 0.61
TPC	305.58 ± 11.38	216.68 ± 5.36
FRAP	2.59 ± 0.06	1.60 ± 0.03
ABTS	2.44 ± 0.15	1.87 ± 0.26

^z^ Mean (n = 3 ± SE). L*: Luminosity (0 to 100). TA (g CE/L): g citric acid equivalent per liter. TPC: mg GAE/L. FRAP: mmol Fe^+2^/L. ABTS: Trolox equivalent antioxidant capacity: mmol TEAC/L.

**Table 3:** Physicochemical parameters of the Jimbee and Okashi melon wines.

Parameter	Jimbee Wine	Okashi Wine
Alcohol (%; *v*/*v*)	9.8 ^z^ ± 0.0	9.9 ± 0.0
TSS (°Brix)	11.30 ± 0.31	8.93 ± 0.07
Residual sugar (g/L)	1.82 ± 0.34	1.67 ± 0.22
Sugar-free extract (g/L)	53.38 ± 2.26	25.33 ± 0.28
pH	4.09 ± 0.02	4.01 ± 0.03
TA (g TE/L)	8.86 ± 0.31	6.88 ± 0.19
Volatile acidity (g/L)	0.38± 0.09	0.21 ± 0.52
SO_2_ free (mg/L)	131.84 ± 2.56	63.15 ± 0.74
SO_2_ total (mg/L)	142.40 ± 1.85	97.60 ± 1.07
L*	50.17 ± 0.21	61.32 ± 0.03
°Hue	95.67 ± 0.37	107.54 ± 0.07
Chroma	13.68 ± 0.15	10.06 ± 0.28
TPC	406.71 ± 3.26	242.17 ± 2.40
FRAP	5.33 ± 0.15	3.07 ± 0.02
ABTS	1.94 ± 0.01	1.04 ± 0.03

^z^ Means (n = 3 ± SE). L*: Luminosity (0 to 100). TA (g CE/L): g citric acid equivalent per liter. TPC: mg GAE/L. FRAP: mmol Fe^+2^/L. ABTS: Trolox equivalent antioxidant capacity: mmol TEAC/L.

**Table 4:** Individual sugar (g/L) of the Jimbee and Okashi melon wines.

Soluble Sugar	Jimbee Wine	Okashi Wine
Glucose	0.04 ^z^ ± 0.00	0.04 ± 0.00
Fructose	1.02 ± 0.20	1.44 ± 0.21
Saccharose	0.63 ± 0.11	LD < 0.01
Maltose	0.13 ± 0.04	0.18 ± 0.01
Total	1.82 ± 0.34	1.66 ± 0.22

^z^ Mean (n = 3 ± SE). Level of detection (LD).

**Table 5:** Identification and quantification (μg/L) of volatile compounds in the Jimbee and Okashi melon musts and wines.

R.T.	Tentative ID	KI	Jimbee Must	Jimbee Wine	Okashi Must	Okashi Wine
	*Esters*					
2.171	Methyl acetate	<1100	117.08 ^z^ ± 1.57	n.d.	n.d.	n.d.
2.477	Ethyl Acetate	<1100	219.19 ± 2.51	672.16 ± 117.71	151.56 ± 2.34	456.61 ± 59.24
3.079	Ethyl propionate	<1100	15.24 ± 0.08	n.d.	19.25 ± 0.32	n.d.
3.276	n-Propyl acetate	<1100	36.87 ± 0.53	n.d.	19.76 ± 0.34	n.d.
3.793	Isobutyl acetate	<1100	132.65 ± 2.13	n.d.	113.95 ± 2.77	n.d.
4.187	Ethyl butanoate	<1100	63.18 ± 0.69	36.10 ± 6.40	41.46 ± 0.77	n.d.
4.471	Ethyl 2-methyl-butanoate	<1100	22.03 ± 0.60	n.d.	48.16 ± 0.80	n.d.
4.876	Butyl acetate	<1100	257.51 ± 5.22	22.66 ± 7.37	87.90 ± 2.06	n.d.
6.047	2-Methyl-1-butyl acetate	<1100	40.69 ± 3.64	194.39 ± 45.14	64.60 ± 3.79	310.69 ± 91.07
6.582	Propyl 2-methyl-butanoate	<1100	n.d.	n.d.	6.08 ± 0.41	n.d.
7.623	Pentyl acetate	1110	n.d.	n.d.	7.42 ± 0.92	n.d.
10.047	Ethyl hexanoate	1194	6.31 ± 0.23	156.95 ± 40.09	7.28 ± 0.11	405.28 ± 68.15
11.861	Hexyl acetate	1245	62.41 ± 1.08	n.d.	63.35 ± 3.68	n.d.
13.790	4-Hexen-1-ol acetate-	1290	n.d.	n.d.	37.76 ± 1.17	n.d.
20.526	Ethyl octanoate	1423	n.d.	1271.05 ± 232.08	n.d.	3876.75 ± 276.55
21.683	Heptyl formate	1445	7.50 ± 0.55	n.d.	n.d.	n.d.
22.693	Octyl acetate	1463	17.02 ± 3.86	n.d.	n.d.	n.d.
23.312	Butane-2,3-diyl diacetate	1474	18.90 ± 0.29	n.d.	n.d.	n.d.
26.111	Ethyl nonanoate	1519	n.d.	27.32 ± 0.87	n.d.	25.20 ± 0.83
28.830	Ethyl 3-Nonenoate	1556	n.d.	n.d.	n.d.	29.31 ± 1.74
29.328	Methyl decanoate	1562	n.d.	n.d.	n.d.	49.65 ± 1.94
31.552	Ethyl decanoate	1590	n.d.	600.12 ± 79.84	n.d.	1197.58 ± 58.97
32.185	3-Methylbutyl octanoate	1597	n.d.	n.d.	n.d.	63.40 ± 5.21
33.515	Phenylmethyl acetate	1695	4.82 ± 2.41	n.d.	6.08 ± 0.06	n.d.
33.005	Ethyl 9-decenoate	1651	n.d.	n.d.	n.d.	696.19 ± 72.37
33.881	Ethyl methoxyacetate	1711	n.d.	49.24 ± 14.90	n.d.	32.89 ± 0.81
35.245	Phenethyl acetate	1761	n.d.	74.92 ± 0.73	n.d.	258.95 ± 3.74
35.354	Methyl laurate	1765	n.d.	31.82 ± 4.70	n.d.	n.d.
36.056	Ethyl laurate	1790	n.d.	318.35 ± 41.66	n.d.	n.d.
36.361	3-Methylbutyl decanoate	1802	n.d.	n.d.	n.d.	41.58 ± 1.58
38.724	Methyl 2,4,6-trimethylnonanoate	2005	n.d.	101.73 ± 2.15	n.d.	n.d.
39.783	Ethyl nonanoate	2102	n.d.	n.d.	n.d.	10.37 ± 1.07
40.420	Methyl palmitate	2159	n.d.	n.d.	n.d.	44.54 ± 0.81
40.827	Ethyl palmitate	2195	n.d.	67.14 ± 12.19	n.d.	377.98 ± 10.05
	*Alcohols*					
3.009	Ethanol	<1100	n.d.	4407.75 ± 133.36	n.d.	6472.94 ± 94.29
4.308	1-Propanol	<1100	n.d.	n.d.	n.d.	19.06 ± 5.84
5.639	Isobutanol	<1100	n.d.	107.13 ± 9.25	n.d.	144.24 ± 4.95
9.057	2-Methyl-1-butanol	1163	38.64 ± 1.14	n.d.	15.40 ± 0.73	n.d.
9.237	3-Methyl-1-butanol	1169	n.d.	1425.50 ± 69.14	n.d.	2800.05 ± 92.86
16.037	1-Hexanol	1339	93.73 ± 2.23	67.06 ± 1.42	7.43 ± 0.73	n.d.
21.383	1-Nonen-3-ol	1439	13.74 ± 1.42	n.d.	n.d.	n.d.
27.558	1-Octanol	1539	96.66 ± 1.03	51.96 ± 1.53	n.d.	n.d.
31.010	Nonen-1-ol isomer	1583	8.73 ± 1.16	n.d.	5.31 ± 1.53	n.d.
32.325	1-Nonanol	1599	14.48 ± 1.07	n.d.	11.45 ± 0.31	n.d.
32.828	Nonen-1-ol isomer	1636	13.71 ± 0.35	n.d.	18.27 ± 1.48	n.d.
33.697	Nonen-1-ol isomer	1705	48.79 ± 4.14	n.d.	45.82 ± 2.67	n.d.
34.295	3,6-Nonadien-1-ol	1727	40.72 ± 0.09	n.d.	n.d.	n.d.
34.702	Citronellol	1742	n.d.	38.99 ± 1.58	n.d.	n.d.
36.210	Benzyl alcohol	1796	29.19 ± 0.67	n.d.	12.38 ± 0.28	344.52 ± 27.24
36.772	Phenylethyl alcohol	1835	6.34 ± 0.28	330.77 ± 15.06	2.04 ± 0.10	648.11 ± 7.16
41.083	2,4-Bis(1,1-dimethylethyl)-phenol	2218	n.d.	356.03 ± 7.23	n.d.	512.36 ± 16.06
	*Unsaturated aliphatic*					
9.240	1-Heptene	1169	n.d.	n.d.	15.94 ± 1.47	n.d.
32.208	6-Methyl-1-Octene	1598	n.d.	35.18 ± 3.19	n.d.	n.d.
39.595	2-Methoxy-2-methylbut-3-ene	2085	n.d.	19.62 ± 5.80	n.d.	n.d.
	*Aldehyde*					
12.465	Octanal	1260	n.d.	n.d.	35.84 ± 0.34	n.d.
18.052	Nonanal	1377	n.d.	n.d.	17.02 ± 1.57	n.d.
21.090	6-Nonenal	1434	n.d.	n.d.	236.10 ± 6.84	n.d.
24.126	Benzaldehyde	1488	n.d.	n.d.	5.51 ± 0.37	n.d.
28.256	2,6-Nonadienal, isomer	1548	16.90 ± 1.81	n.d.	97.13 ± 4.07	n.d.
	*Fatty acids*					
20.714	Acetic acid	1427	n.d.	701.42 ± 15.40	n.d.	314.59 ± 9.67
35.914	Hexanoic acid	1785	n.d.	n.d.	n.d.	78.13 ± 2.74
38.621	Octanoic acid	1995	n.d.	118.19 ± 14.62	n.d.	509.21 ± 18.20
	*Miscellaneous*					
10.934	3-Octanone	1220	7.83 ± 1.07	n.d.	5.99 ± 0.20	n.d.
19.754	Bis(1,1-dimethylethyl)-benzene isomer	1408	36.68 ± 2.54	152.42 ± 8.11	n.d.	193.27 ± 9.13
22.620	S-(3-Hydroxypropyl) thioacetate	1462	n.d.	n.d.	13.94 ± 2.81	n.d.
30.469	3-(Methylthio)propyl acetate	1577	35.18 ± 1.15	n.d.	14.88 ± 0.66	n.d.
37.142	1-Ethenyl-2-methylbenzene	1863	n.d.	n.d.	n.d.	48.13 ± 2.02
	TOTAL		2145.56 ± 56.55	12,788.44 ± 1.228.65	1769.79 ± 26.15	20,504.38 ± 1.477.05

^z^ Mean (n = 3 ± SE). R.T.: Retention time. KI: Kovats Index. n.d.: Not detected.

**Table 6:** Volatile compounds identified with OAV > 1 and its ROC for the Jimbee and Okashi melon wines.

Tentative ID	Threshold (mg/L)	Odor Description	OAV		ROC	
			Jimbee	Okashi	Jimbee	Okashi
Ethyl butanoate ^a^	20	Pineapple, apple	1.81 ^z^	<1	0.06	<0.01
Ethyl hexanoate ^b^	8	Fruity, green, wine-like	19.62	50.66	0.68	0.44
Ethyl octanoate ^a^	580	Sweet, flora, fruity, pear	2.19	6.68	0.08	0.06
Ethyl decanoate ^a^	200	Floral, fruity,	3.00	5.99	0.10	0.05
Ethyl 9-decenoate ^b^	100	Rose	<1	6.96	<0.01	0.06
Phenethyl acetate ^c^	250	Floral, honey, rose	<1	1.04	0.01	0.01

^z^ Mean (n = 3 ± SE). ^a, b, c^ Thresholds references: [18,19,20]. OAV: odor activity value. ROC: relative odor contribution.

**Table 7:** Sensory evaluation of the Jimbee and Okashi melon wines.

Organoleptical Characteristics	Jimbee Wine	Okashi Wine	Range (Excellent to Inadequate)
**Visual**	**7.33 ^z^ ± 1.82**	**13.67 ± 0.61**	**(15–3)**
Limpidity	2.00 ± 0.68	4.67 ± 0.21	(5–1)
Aspect other than limpidity	5.33 ± 1.23	9.00 ± 0.45	(10–2)
**Nose**	**22.5 ± 1.61**	**23.83 ± 2.12**	**(30–12)**
Genuineness	3.83 ± 0.31	5.33 ± 0.21	(6–2)
Positive intensity	6.33 ± 0.56	6.50 ± 0.56	(8–2)
Quality	12.33 ± 0.95	12.00 ± 1.71	(16–8)
**Taste**	**29.83 ± 2.55**	**35.5 ± 0.96**	**(44–18)**
Genuineness	3.67 ± 0.56	4.17 ± 0.48	(6–2)
Positive intensity	4.83 ± 0.75	6.83 ± 0.17	(8–2)
Harmonious persistence	5.83 ± 0.48	6.50 ± 0.34	(8–4)
Quality	15.50 ± 1.20	18.00 ± 0.63	(22–10)
**Harmony—Overall judgement**	**8.50 ± 0.50**	**9.83 ± 0.17**	**(11–7)**
**TOTAL**	**68.17 ± 3.22**	**82.83 ± 1.96**	**(100–40)**

^z^ Mean (n = 12 ± SE).

## Data Availability

The datasets supporting the findings of this article are available upon reasonable request.
